# IL‐4Rα‐expressing CD11c^+^ cells contribute to driving optimal cellular responses during *Schistosoma mansoni* infection in mice

**DOI:** 10.1002/JLB.MA0318-115R

**Published:** 2018-11-30

**Authors:** Hlumani Ndlovu, Justin Komguep Nono, Natalie Eva Nieuwenhuizen, Frank Brombacher

**Affiliations:** ^1^ Department of Integrative Biomedical Sciences Faculty of Health Sciences University of Cape Town Cape Town South Africa; ^2^ International Center for Genetic Engineering and Biotechnology (ICGEB) Cape Town Component Cape Town South Africa; ^3^ Division of Immunology Institute of Infectious Diseases and Molecular Medicine (IDM) University of Cape Town Cape Town South Africa; ^4^ Immunology of Infectious Disease Research Unit South African Medical Research Council (SAMRC) Cape Town South Africa; ^5^ The Medical Research Centre Institute of Medical Research and Medicinal Plant Studies (IMPM) Ministry of Scientific Research and Innovation Yaoundé Cameroon

**Keywords:** acute, CD11c^+^ cells, IL‐4Rα, immunity, mice, schistosomiasis

## Abstract

Development of IL‐4 receptor alpha (IL‐4Rα)‐dependent cellular immunity regulates host protection against acute schistosomiasis. In this study, we investigated the importance of IL‐4Rα‐expressing CD11c^+^ cells in driving the development of optimal cellular responses to *Schistosoma mansoni* infection by using CD11c^cre^IL‐4Rα^−/lox^ BALB/c mice, which lacked IL‐4Rα expression on dendritic cells and alveolar macrophages. Abrogation of IL‐4Rα expression on CD11c^+^ cells affected activation of CD4^+^ T cells, resulting in reduced numbers of effector CD4^+^ T cells and impaired production of Th1 and Th2 cytokines by CD4^+^ T cells ex vivo. However, secretion of both type 1 and type 2 Ab isotypes was unchanged in infected CD11c‐specific IL‐4Rα‐deficient mice compared to littermate controls. Together, these data demonstrate that IL‐4Rα‐expressing CD11c^+^ cells play an important role in maintaining cellular immunity during schistosomiasis in mice.

**Abbreviations**
AST: aspartate transaminase
BMDCs: bone marrow‐derived dendritic cells
DC: dendritic cell
IL‐4Rα: IL‐4 receptor alpha
MLN: mesenteric lymph node
pLN: popliteal lymph node
SEA: schistosome egg antigen Ag

## 1 INTRODUCTION

Dendritic cells (DCs) are the most specialized APCs, capable of recognizing and processing foreign Ag, migrating to the lymph nodes, and efficiently initiating T cell activation.^1^, ^2^, ^3^ The ability of DCs to orchestrate Th1/Th17 immunity in response to bacterial and viral pathogens is well established, whereas there is a paucity of understanding about their activation and functional capability in a Th2 disease setting.^4^, ^5^ However, recent studies, conducted using CD11c‐diphtheria toxin receptor mice to deplete CD11c^+^ DCs, have demonstrated a critical role for CD11c^+^ DCs in inducing Th2 immunity during *Schistosoma mansoni* infection.^6^ Furthermore, bone marrow‐derived dendritic cells (BMDCs) have been shown to respond to IL‐4 by up‐regulating alternative activation markers such as Ym1/2 and RELMα, and abrogation of IL‐4 receptor alpha (IL‐4Rα) expression on DCs resulted in impaired IFN‐γ production in both Th1 and Th2 settings.^7^ Interestingly, IL‐4 has been shown to instruct BMDCs to produce IL‐12p70 in the presence of bacterial LPS or CpG in vitro.^8^, ^9^, ^10^, ^11^ The instruction theory was shown to be relevant in vivo because the treatment of susceptible BALB/c mice with IL‐4 conferred host protection to *Leishmania major* infection.^8^ Recently, it was shown that DCs from mice carrying a specific deletion of IL‐4Rα on CD11c^+^ cells had reduced IL‐12p40 expression and increased IL‐10 expression during *L. major* infection, coinciding with impaired Th1‐type responses and severe disease characterized by footpad necrosis.^12^ Furthermore, IL‐4Rα expression on CD11c^+^ cells was shown to promote allergen‐induced Th2 effector responses in allergic airway disease in mice.^13^ DCs IL‐4Rα expression therefore appears to play an important role in both Th1‐ and Th2‐associated diseases.

Schistosomiasis is an important parasitic disease that infects more than 200 million people, mostly in developing countries, and causes an estimated 280,000 deaths per annum in sub‐Saharan Africa alone.^14^, ^15^ During infection in both humans and mice, *S. mansoni* eggs trapped in the host tissue (particularly in the liver) induce a strong granulomatous inflammatory response that is accompanied by augmented production of Th2 cytokines, eosinophilia, goblet cell hyperplasia, and increased IgG1 and IgE production.^5^, ^16^, ^17^ Infection of IL‐4^−/−^ and IL‐4/IL‐13^−/−^ mice with *S. mansoni* revealed a crucial protective role for Th2 cytokine and granuloma formation.^18^, ^19^, ^20^ IL‐4 and IL‐13 are key Th2 cytokines that signal though the common receptor chain, the IL‐4Rα.^21^, ^22^ IL‐4 exclusively signals through the type 1 receptor composed of IL‐4Rα and the common gamma chain, while both IL‐4 and IL‐13 signal through the type II receptor comprising of IL‐4Rα and IL‐13Rα1 chains.^21^, ^22^ Mice deficient in IL‐4Rα signaling quickly succumb to *S. mansoni* infection due to impaired granuloma formation, impaired Th2 polarization, increased liver inflammation, and destruction of the gut integrity that results in endotoxemia and septic shock.^18^, ^23^, ^24^ Therefore, IL‐4 and IL‐13 responsiveness plays a key role in down‐regulating organ injury induced by *S. mansoni* eggs.

Generation of cell‐specific IL‐4Rα deficient mouse strains has proven to be an invaluable tool for dissecting the mechanisms conferring host protection or susceptibility to *S. mansoni* infection. Previous studies from our laboratory have demonstrated a pivotal role for IL‐4Rα expressing macrophages in host protective responses during acute schistosomiasis.^24^ Mice deficient in IL‐4Rα expression on macrophages (LysM^cre^IL‐4Rα^−/lox^) were extremely susceptible to *S. mansoni* infection due to increased hepatocellular damage and intestinal inflammation that led to endotoxemia and septic shock.^25^ Furthermore, using mice that lacked IL‐4Rα expression on pan‐T cells (iLck^cre^IL‐4Rα^−/lox^), we showed that IL‐4/IL‐13 responsive non‐CD4^+^ T cells contribute to resistance against acute schistosomiasis by controlling excessive liver inflammation.^23^ In contrast, mice deficient in IL‐4Rα expression specifically on CD4^+^ T cells (Lck^cre^IL‐4Rα^−/lox^) survived *S. mansoni* infection despite augmented Th1 granulomatous pathology.^26^ Other possible IL‐4/IL‐13 responsive cellular populations contributing to the mechanism of host resistance to *S. mansoni* infection are yet to be identified. Therefore, we investigated whether IL‐4Rα signaling on CD11c^+^ cells plays a role in orchestrating cellular responses and host protection to *S. mansoni* infection, by utilizing a transgenic mouse strain (CD11c^cre^IL‐4Rα^−/lox^) that lacked IL‐4Rα expression specifically on CD11c^+^ cells.^12^ We found that effector CD4^+^ T cells and B cells numbers were drastically reduced in the draining mesenteric lymph nodes (MLNs) of CD11c^cre^IL‐4Rα^−/lox^ mice compared to littermate controls. Together, these data demonstrate that IL‐4Rα signaling on CD11c^+^ cells is important for driving cellular immunity during acute schistosomiasis.

## 2 RESULTS

### 2.1 Impaired Th1 and Th2 cytokine responses in CD11^cre^IL‐4Rα^−/lox^ mice challenged with *S. mansoni* eggs in the footpad

**Figure 1:**
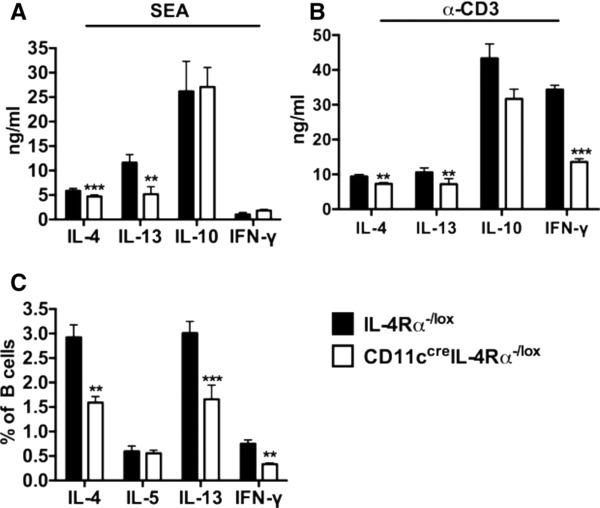
**Impaired Th1 and Th2 cytokine responses in CD11c^cre^IL‐4R^−/lox^ mice challenged with *S. mansoni* eggs in a footpad**. IL‐4Rα^−/lox^ and CD11c^cre^IL‐4Rα^−/lox^ mice were challenged with 2,500 *S. mansoni* eggs and killed 7 d postinfection to harvest pLN. Single cell suspensions were prepared from pLN and total cells were restimulated with either 20 μg/ml SEA or 20 μg/ml α‐CD3, and cytokine secretion was detected by ELISA. (**A** and **B**) Detection of cytokines by ELISA after restimulation of total pLN cells with either SEA or α‐CD3. (C) Frequency of CD19^+^ B cells secreting cytokines after restimulation of total pLN cells with 50 ng/ml PMA and 250 ng/ml Ionomycin in the presence of 200 μM monensin. Data are representative of 2 independent experiments; *n* = 6 mice. *P < 0.05 and **P < 0.01 vs. IL‐4Rα^−/lox^ mice using a two‐way ANOVA with Bonferroni's posttest

To determine the effect of CD11c^+^ cell IL‐4Rα expression on Th1/Th2 type responses to *S. mansoni*, we challenged CD11c^cre^IL‐4Rα^−/lox^ and IL‐4Rα^−/lox^ littermate control mice with 2,500 *S. mansoni* eggs in the left hind footpad and collected popliteal lymph nodes (pLNs) 7 d postchallenge. We stimulated total pLN cells with either Schistosome egg Ag (SEA) or α‐CD3 and analyzed cytokine secretion by sandwich ELISA. Secretion of IL‐4 and IL‐13 was reduced in total pLN cells from CD11c^cre^IL‐4Rα^−/lox^ mice compared to control mice after either Ag‐specific or mitogenic restimulation (Fig. 1A and B), despite unaltered frequencies of CD19^+^ B cells or CD4^+^ T cells (Supplementary Fig. S1A and B). Moreover, production of the Th1 cytokine IFN‐γ was reduced in α‐CD3‐stimulated cells from CD11c^cre^IL‐4Rα^−/lox^ mice compared to control mice (Fig. 1B). Little or no cytokine expression was detected in naïve mutant or control mice after antigenic or mitogenic restimulation (Fig. S2). We also found that the frequency of CD19^+^ B cells secreting IL‐4, IL‐13, and IFN‐γ after stimulation with PMA/Ionomycin ex vivo was reduced in CD11c^cre^IL‐4Rα^−/lox^ mice compared to littermate control mice (Fig. 1C, Supplementary Fig. S3A). Together, these data suggest that specific deletion of IL‐4Rα on CD11c^+^ cells impairs secretion of both Th1 and Th2 cytokines during synchronous eggs challenge in the footpad.

### 2.2 Reduced cellular immune responses in *S. mansoni‐*infected CD11^cre^IL‐4Rα^−/lox^ mice

**Figure 2:**
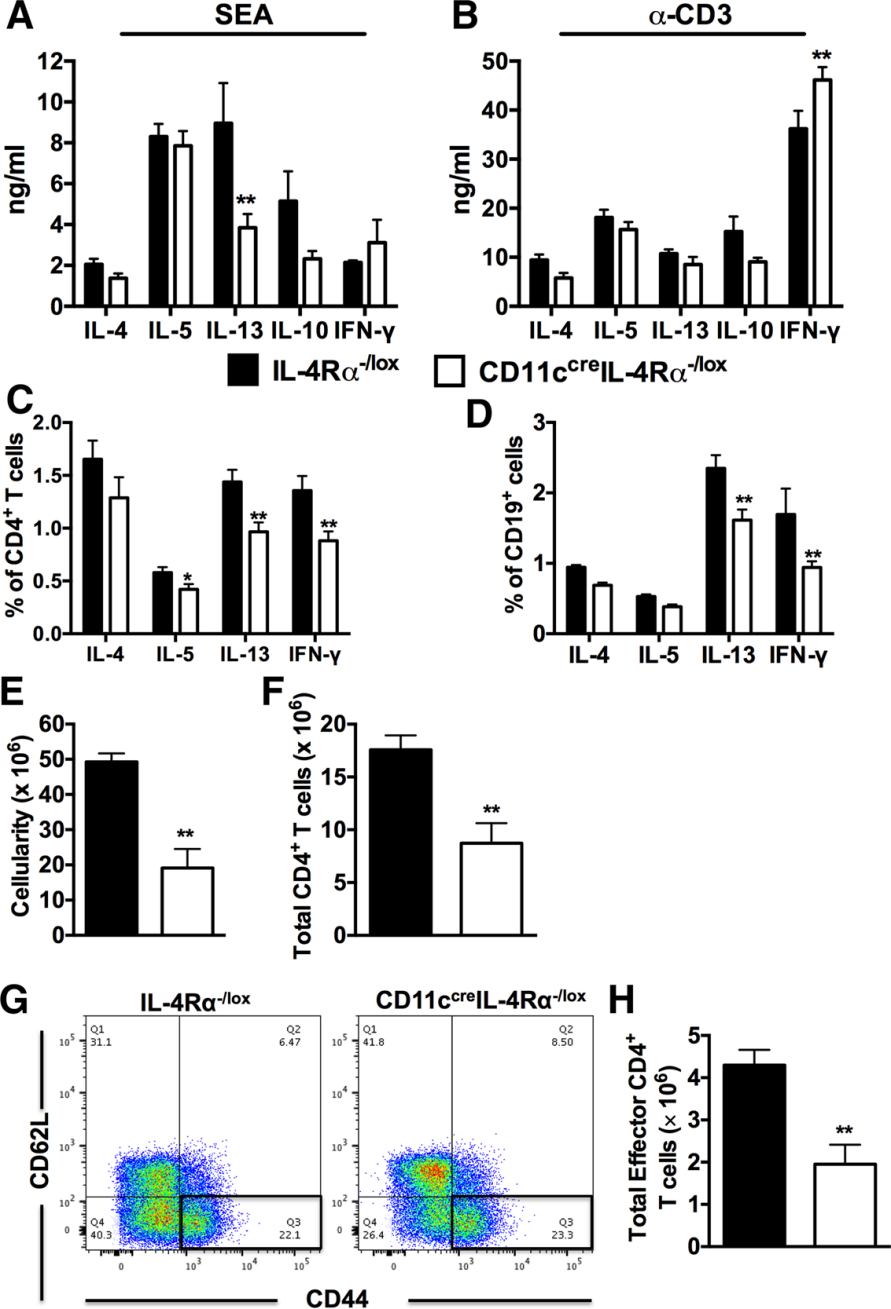
**Decreased numbers of CD4^+^ T cells recruited to the draining lymph node of infected CD11c^cre^IL‐4Rα^−/lox^ mice**. IL‐4Rα^−/lox^ and CD11c^cre^IL‐4Rα^−/lox^ mice were infected with 100 live *S. mansoni* cercariae and killed 7 wk postinfection. Single cell suspension was prepared from MLN and cells were stained for flow cytometry. (**A** and **B**) Cytokine production by total MLN cells restimulated with either SEA or α‐CD3 (mean ± sem) ex vivo was analyzed by ELISA. (**C** and **D**) Intracellular cytokine production by MLN CD4^+^ T cells and CD19^+^ B cells restimulated with 50 ng/ml PMA and 250 ng/ml Ionomycin ex vivo in the presence of 200 μM monensin. (**E**) Total number of cell number in the draining lymph node. *P < 0.05 and **P < 0.01 using a two‐way ANOVA with Bonferroni's posttest. (**F**) Total numbers of CD4^+^ T cells. (**G** and **H**) FACS plots showing gating of effector CD4^+^ T cells and total number of effector CD4^+^ T cells (CD4^+^CD44^hi^CD62L^lo^) in the draining lymph node. Data are representative of 2 independent experiments; *n* = 6 mice. *P < 0.05 and **P < 0.01 using two‐tailed Mann–Whitney nonparametric Student's *t*‐test

Immunity to *S. mansoni* eggs is characterized by development of IL‐4Rα‐dependent Th2 immune responses and granuloma formation.^23^, ^24^ To investigate the impact of deleting IL‐4Rα expression on CD11c^+^ cells, we infected CD11c^cre^IL‐4Rα^−/lox^ mice and littermate control mice with 100 live *S. mansoni* cercariae and assessed immune responses at 7 wk postinfection. We prepared single cell suspensions from harvested MLN, analyzed cytokine responses after restimulation with either SEA or α‐CD3, and used flow cytometry to analyze cell populations that were recruited to the MLN during infection. Impaired IL‐4Rα expression on CD11c^+^ cells did not alter the production of IL‐4, IL‐5, and IL‐10 after Ag‐specific stimulation (SEA) of total MLN cells, except for reduced IL‐13 production compared to littermate control mice (Fig. 2A). Similarly, the production of Th2 cytokines was not altered after stimulation with α‐CD3 in CD11c^cre^IL‐4Rα^−/lox^ mice compared to littermate control mice except for increased production of IFN‐γ (Fig. 2B). Cytokine secretion by MLN CD4^+^ T cells was also determined by intracellular flow cytometry. Although the percentage of CD4^+^ T cells secreting IL‐4 was similar between CD11c^cre^IL‐4Rα^−/lox^ mice and littermate controls (Supplementary Fig. S1D), the percentage of CD4^+^ T cells producing IL‐5, IL‐13, and IFN‐γ was significantly reduced in CD11c^cre^IL‐4Rα^−/lox^ mice compared to littermate control mice (Fig. 2C, Supplementary Fig. S3B). Moreover, the frequency of CD19^+^ B cells secreting IL‐4, IL‐5, IL‐13, and IFN‐γ was significantly reduced in CD11c^cre^IL‐4Rα^−/lox^ mice compared to littermate control mice (Fig. 2D), despite an unaltered frequency of CD19^+^ B cells between the infected mutant strains (Supplementary Fig. S3C). Together, these data suggest that IL‐4Rα‐expressing CD11c^+^ cells play a role in initiating and maintaining cytokine production by CD4^+^ T cells in vivo.

We also analyzed the numbers of lymphocytes in the MLN after infection using flow cytometry. We found that the total number of MLN cells was significantly reduced (60%) in CD11c^cre^IL‐4Rα^−/lox^ mice compared to IL‐4Rα^−/lox^ littermate control mice at 7 wk postinfection (Fig. 2E). This corresponded with a striking decrease in the total number of CD4^+^ T cells (Fig. 2F) and effector CD4^+^ T cells (Fig. 2G and H) in CD11c^cre^IL‐4Rα^−/lox^ mice compared to littermate control mice. Therefore, these data demonstrate that IL‐4Rα‐expressing CD11c^+^ cells are required for expansion of CD4^+^ T cells in the MLN during *S. mansoni* infection.

### 2.3 Production of specific IgG and total IgE during *S. mansoni* infection is unaffected by deletion of IL‐4Rα on CD11c^+^ cells

**Figure 3:**
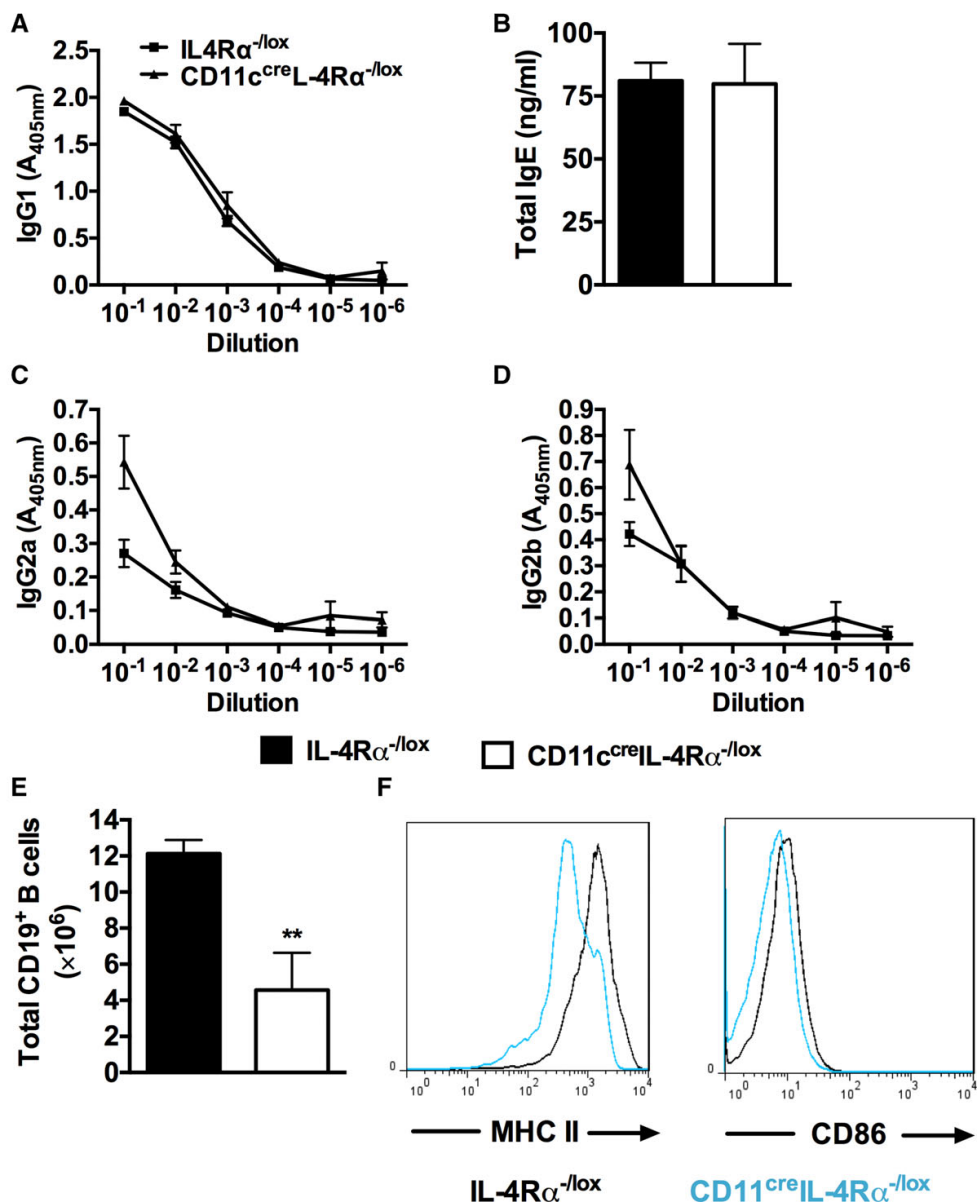
**Ab responses in infected CD11c^+^ cell‐specific IL‐4Rα deficient mice were similar to littermate controls**. IL‐4Rα^−/lox^ and CD11c^cre^IL‐4Rα^−/lox^ mice were infected with 100 live *S. mansoni* cercariae and analyzed 7 wk postinfection. (**A**–**D**) Serum Ab titers for SEA‐specific immunoglobulin (IgG1, IgG2a, and IgG2b) and total IgE were quantified by ELISA. (**E**) Total number of CD19^+^ B cells in the MLN. (**F**) Histograms showing the expression of MHCII and CD86 by CD19^+^ B cells. Data represents 2 independent experiments; *n* = 4–6 mice. *P < 0.05, **P < 0.01, and ***P < 0.001 using two‐tailed Mann–Whitney nonparametric Student's *t*‐test

Depletion of CD11c^+^ DCs has been shown to affect B cell development during *S. mansoni* infection.^6^ To investigate the impact of impairing IL‐4Rα signaling specifically on CD11c^+^ cells, Ag‐specific Ab isotypes (IgG1, IgG2a, and IgG2b) and total IgE were determined by ELISA. Abrogation of IL‐4Rα expression on CD11c^+^ cells did not impair isotype class switching, as shown by the similar levels of type 2 Ab isotypes (SEA‐specific IgG1 and total IgE) and type 1 Ab isotypes (SEA‐specific IgG2a and IgG2b) in CD11c^cre^IL‐4Rα^−/lox^ and littermate control mice (Fig. 3A–D). There was a dramatic decrease in the number of CD19^+^ B cells in the MLN of CD11c^cre^IL‐4Rα^−/lox^ mice compared to littermate control mice (Fig. 3E, Supplementary Fig. S2), related to the reduced cellularity in the lymph node compared to littermate control mice as shown above (Fig. 2E). Expression of the activation markers MHCII and CD86 was reduced on CD19^+^ B cells of CD11c^cre^IL‐4Rα^−/lox^ mice compared to littermate control mice (Fig. 3F). Together, these data suggest that IL‐4Rα‐expressing CD11c^+^ cells contribute to the expansion and activation of B cells in the draining lymph node during *S. mansoni* infection.

### 2.4 CD11c^cre^IL‐4Rα^−/lox^ mice show reduced perioval liver fibrogranulomatous inflammation after *S. mansoni* infection

**Figure 4:**
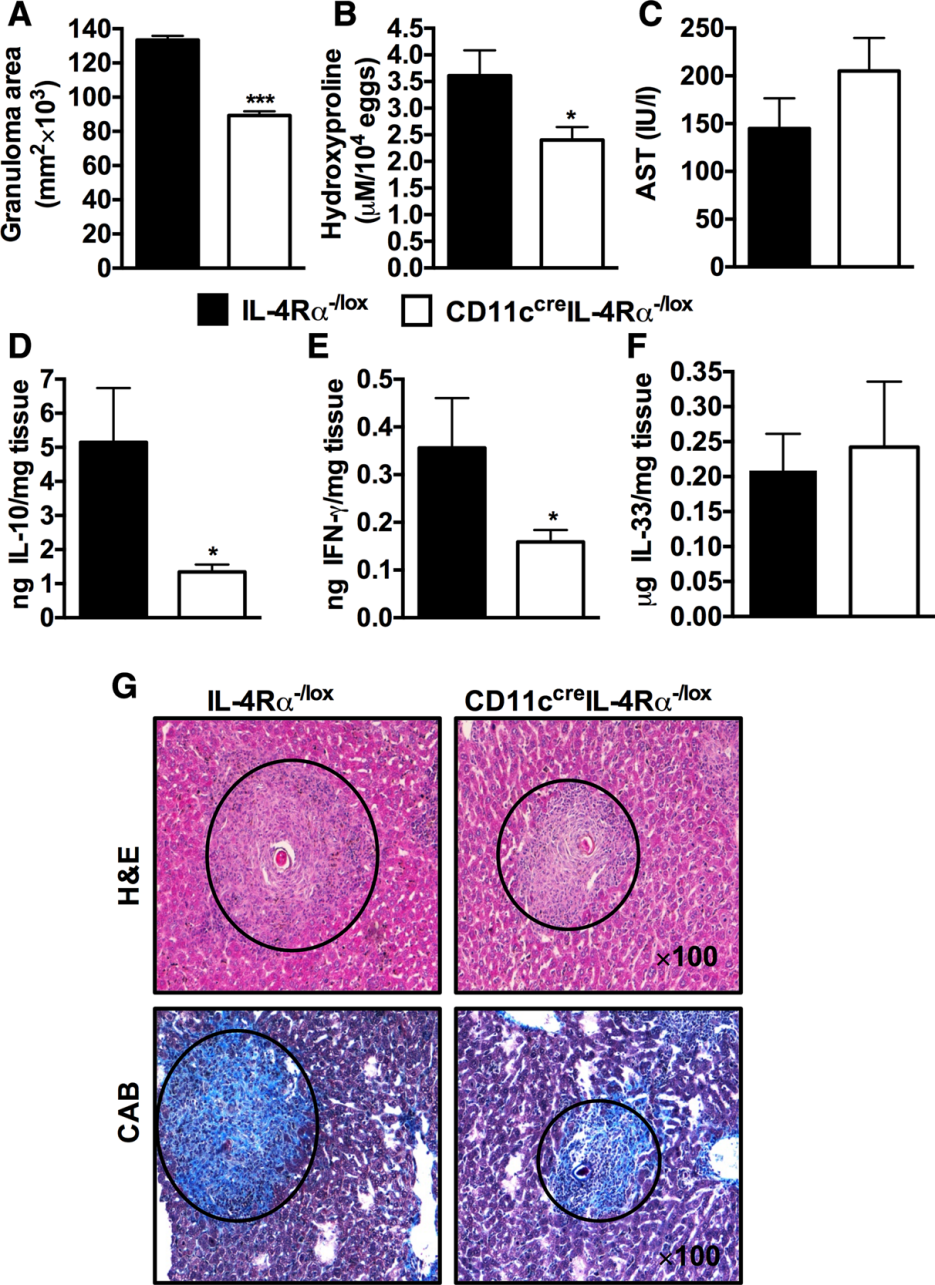
**CD11c^cre^IL‐4Rα^−/lox^ mice show reduced liver pathology during *S. mansoni* infection**. IL‐4Rα^−/lox^ and CD11c^cre^IL‐4Rα^−/lox^ mice were infected with 100 live *S. mansoni* cercariae and analyzed 7 wk postinfection. (**A**) Granuloma area surrounding eggs quantified by microscopic analysis on H&E‐stained sections. Twenty to 30 granulomas per mouse were included in the analysis. (**B**) Liver fibrosis measured as hydroxyproline normalized to egg numbers. (**C**) Hepatocellular damage measured as serum levels of aspartate transaminase enzyme. (**D**–**F**) Liver was homogenized, protein content determined using Pierce BCA assay kit, cytokines detected by ELISA, and normalized to tissue weight. (**G**) Formalin‐fixed liver sections (100×) stained with H&E for morphological analysis or CAB for collagen content. Data are representative of 2 independent experiments; *n* = 4–6 mice. *P < 0.05, **P < 0.01, and ***P < 0.001 using two‐tailed Mann–Whitney nonparametric Student's *t*‐test

A major site for egg entrapment during schistosomiasis is the liver, where a considerable part of the fibrogranulomatous inflammation takes place.^24^ Therefore, we investigated the contribution of IL‐4Rα‐expressing CD11c^+^ cells in modulating granulomatous liver inflammation in mice. Interestingly, abrogation of IL‐4Rα signaling on CD11c^+^ cells resulted in smaller granulomas (Fig. 4A), reduced liver fibrosis, indicated by reduced hydroxyproline concentrations (Fig. 4B), and normal serum aspartate transaminase (AST) levels compared to infected IL‐4Rα^−/lox^ littermate control mice (Fig. 4C). We found comparable egg burdens in the liver and small intestines in both strains of infected mice (Supplementary Fig. S4), suggesting that the progression of infection was similar. Moreover, CD11c^cre^IL‐4Rα^−/lox^ mice had reduced levels of tissue IL‐10 and IFN‐γ and unaltered levels of tissue IL‐33 compared to littermate control mice (Fig. 4D–F). Histological examination of liver sections confirmed that CD11c^cre^IL‐4Rα^−/lox^ mice developed smaller granulomas around parasite eggs than the littermate control mice (Fig. 4F). We found comparable levels of IL‐10, IFN‐γ, and IL‐33 in the small intestines between mutant mice (Supplementary Fig. S5A–C). Finally, we observed comparable gut pathology in both the infected mutant mice during acute schistosomiasis (Supplementary Fig. S5D). Therefore, these data suggest that IL‐4Rα‐expressing CD11c^+^ cells partake in driving fibrogranulomatous perioval liver inflammation during schistosomiasis.

Previously, it was shown that BMDCs restimulated with recombinant IL‐4 in vitro up‐regulated the production of Relm‐α and Ym‐1/2 and showed increased mRNA transcripts for *Retnla*, *Clec7a*, *Mrc*1, and *Ccl24*,^7^ markers associated with alternatively activated macrophages^27^, ^28^; hence, we assessed the impact of IL‐4Rα deficiency on CD11c^+^ cells on the phenotype of both CD11c^+^MHCII^+^ and CD11b^+^MHCII^+^ cells from infected mice. CD11c^+^MHCII^+^ DCs from infected CD11c^cre^IL‐4Rα^−/lox^ mice had unaltered Ym‐1 expression compared to littermate control mice while iNOS expression was significantly up‐regulated (Supplementary Fig. S6A and B). However, CD11b^+^MHCII^+^ cells from CD11c^cre^IL‐4Rα^−/lox^ mice displayed a classical activation profile indicated by reduced Ym‐1 expression and increased iNOS expression compared to littermate control mice (Supplementary Fig. S6C and D). We also investigated whether impairing IL‐4Rα expression on DCs altered their ability to produce IL‐12p70 and IL‐10 production. Intracellular flow cytometric analysis revealed that CD11c^+^MHCII^+^ cells from CD11c^cre^IL‐4Rα^−/lox^ mice exhibited a trend toward reduced production of IL‐12p70 and IL‐10 compared to littermate control mice, although the differences were not significant (Supplementary Fig. S6E). Despite the increased frequency of eosinophils (CD11c^−^Siglec‐F^+^; Supplementary Fig. S6F), the proportions of neutrophils (Supplementary Fig. S6H) as well as the number of both eosinophils (Supplementary Fig. S6G) and neutrophils (Fig. S6I) were comparable between both infected strains in the liver. Together, these data suggest that deleting IL‐4Rα expression on CD11c^+^ cells may affect their activation status during *S. mansoni* infection.

## 3 DISCUSSION

CD11c‐expressing cells include DCs, which are important Ag presenters required to educate and activate naïve T cells during infection.^1^, ^3^ Prior studies aimed at elucidate the contribution of CD11c^+^ DCs on the development of sufficient Th2 immune responses during *S. mansoni* infection, relied on depleting CD11c^+^ cells by administering diphtheria toxin in CD11c‐diphtheria toxin receptor mice.^6^ This study demonstrated that depleting CD11c^+^ cells tilted the immune balance toward Th1 immunity by up‐regulating the production of IFN‐γ, while simultaneously impairing the production of Th2 cytokines after synchronous *S. mansoni* eggs challenge in the footpad or during active infection.^6^ A subsequent study from the same group showed that CD11c^+^ DCs respond to IL‐4 by up‐regulating multiple markers that were associated with alternative activation of macrophage and that DCs expressing IL‐4Rα driving optimal production of IFN‐γ in both Th1 and Th2 settings, via a feedback loop in which IL‐4 further instructs DC to produce IL‐12.^7^ We have previously shown that IL‐4Rα‐expressing DCs are crucial for rendering host protection to *L. major* infection^12^ and house dust mite induced allergic asthma.^29^ In this study, we report on the direct contribution of IL‐4Rα‐expressing CD11c^+^ cells in orchestrating optimal cellular immunity to *S. mansoni* infection and modulating tissue granulomatous pathology in vivo.

Previously, we found that T cells of CD11c^cre^IL‐4Rα^−/lox^ mice show the same ability to proliferate and differentiate as T cells of littermate control mice ex vivo.^29^ However, *S. mansoni*‐infected CD11c^cre^IL‐4Rα^−/lox^ mice had a striking reduction of up to 40% in the number of CD4^+^ T cells in the MLN compared to littermate control mice, while the number of effector CD4^+^ T cells was reduced by up to 60%. In addition, CD4^+^ T cells showed reduced secretion of Th2 cytokines IL‐5 and IL‐13 as well as Th1 cytokine IFN‐γ, demonstrated by intracellular FACS and IL‐13 production after Ag‐specific restimulation. Interestingly, when we challenged CD11c^cre^IL‐4Rα^−/lox^ mice with *S. mansoni* eggs in the left hind footpad, we found general impairment of cellular immunity indicated by reduced production of IL‐4, IL‐13, and IFN‐γ after Ag‐specific or mitogenic restimulation. These findings are in line with previous findings from our group and others that have demonstrated an essential role of CD11c^+^ DCs in driving Th2 cytokine production in a Th2 disease setting.^6^, ^7^, ^29^ Therefore, these data suggest that CD11c^+^ cells are crucial for driving optimal cellular immunity during schistosomiasis in mice. We observed a more pronounced effect on cytokine production in CD11c^cre^IL‐4Rα^−/lox^ mice after synchronous eggs challenge in the footpads compared to an active *S. mansoni* infection. Together, these data suggest that CD11c^+^ cells play a key role early during infection to educate T cells to produce both Th1 and Th2 cytokines during *S. mansoni* infection. The complexity of an active infection seems to mask some of these effects.

The effect of interfering with IL‐4Rα expression on CD11c^+^ cells had an even larger impact on the number of CD19^+^ B cells in the MLN, with up to a 70% reduction in the number of CD19^+^ B cells in the MLN. These B cells showed reduced surface expression of the activation markers MHCII and CD86 and produced less IL‐13 and IFN‐γ. These findings expand upon previously published data, where depletion of all CD11c^+^ DCs inhibited B cell development by altering the proportions of marginal zone, follicular, and transitional B cells during the initiation of a response to *S. mansoni* infection,^6^ suggesting that IL‐4Rα expression on CD11c^+^ cells is particularly important in driving these responses. The expression of MHCII on B cells was shown to be essential for the development of sufficient cell‐mediated immunity to *Heligomosomoides polygyrus* infection using mixed bone marrow chimera mice that lacked specific expression of MHCII on B cells.^30^ Moreover, the protective effect offered by B cells expressing MHCII was shown to be independent of Ab production.^30^ In our study, too, Ab responses were unaffected in mice lacking CD11c cells expressing IL‐4Rα, yet our data suggest that IL‐4Rα‐expressing CD11c^+^ cells are important for optimal B cell responses in the gut‐draining lymphatic tissue.

As expected, infected CD11c^cre^IL‐4Rα^−/lox^ mice had reduced tissue granulomatous pathology indicated by reduced granuloma size and fibrosis compared to littermate control mice, in line with the observed impaired Th2 immunity. It is an established fact in the literature that Th2 cytokines, particularly IL‐4 and IL‐13, are critical for liver granuloma formation.^18^, ^23^, ^24^, ^31^, ^32^, ^33^, ^34^ Moreover, IL‐13 has been shown to be a master cytokine mediating fibrogenesis in tandem with other type 2 cytokines during schistosomiasis.^35^, ^36^ In support of this, we consistently found significantly reduced production of IL‐13 during both synchronous *S. mansoni* eggs footpad challenge and active infection in CD11c^cre^IL‐4Rα^−/lox^ mice compared to littermate control mice. This further supports the central role of Th2 cytokines and IL‐4Ra mediated signaling in regulating liver fibrogranulomatous inflammation during schistosomiasis. This aligns with our recent study, which showed that temporal interference with IL‐4Rα expression by oral administration of tamoxifen in i^Cre‐/+^IL‐4Rα^−/lox^ mice during experimental schistosomiasis reduces perioval granulomatous inflammation, tissue fibrosis, scarring, and hepatosplenomegaly.^34^ Our observation of a reduced granulomatous inflammation in CD11c^cre^IL‐4Rα^−/lox^ mice therefore suggests that IL‐4Rα‐expressing CD11c^+^ cells may be contributing to the fibrogranulomatous tissue inflammation in *S. mansoni*‐infected mice.

IL‐4/IL‐13 unresponsive CD11c^+^ cells were found to have unaltered expression of Ym‐1 but increased iNOS expression following *S. mansoni* infection, indicating a subtle shift toward classical activation. These findings are in accordance with a previous study, where it was shown that in vitro stimulation of IL‐4/IL‐13 responsive BMDCs with recombinant IL‐4 resulted in substantial up‐regulation of mRNA transcripts and protein secretion of Relmα, Ym‐1, MR, and Dectin‐1,^7^ markers previously associated with alternatively activated macrophages.^27^, ^28^ Expression of Relm‐α by DCs is also required to prime expression of IL‐10 and IL‐13 by CD4^+^ T cells in a Th2 setting.^7^ In contrast, lymph node DCs from CD11c^cre^IL‐4Rα^−/lox^ mice suffering from cutaneous leishmanisis caused by *L. major* infection showed a shift toward alternative activation with reduced expression of iNOS but increased expression of Arginase‐1 compared to infected littermate control mice.^12^ CD11c^cre^IL‐4Rα^−/lox^ BALB/c mice were found to be hypersusceptible to *L. major* infection due to up‐regulated Th2 cytokine responses, increased type 2 Ab production, and impaired classical activation of macrophages.^12^ This was due to reduced IL‐12 and increased IL‐10 production by IL‐4Rα‐unresponsive DCs from *L. major*‐infected mice.^12^ These results confirmed and extended IL‐4 instruction of DCs to produce IL‐12p70 in the presence of LPS or CpG in vitro or in vivo during *L. major* infection in BALB/c mice.^8^ In this study, we observed a trend toward reduced IL‐10 and IL‐12 expression by CD11c^+^MHCII^+^ cells suggesting that the instruction theory may be irrelevant in the context of helminth infections.

In summary, we found that deleting IL‐4Rα expression on CD11c^+^ cells down‐modulates fibrogranulomatous perioval liver inflammation. This coincided with reduced production of Th2 cytokines particularly IL‐13, reduced number of CD4^+^ T cells, and effector CD4^+^ T cells draining into MLN in infected CD11c^cre^IL‐4Rα^−/lox^ mice compared to control mice. Moreover, B cell responses were significantly abrogated in mice lacking IL‐4Rα expression on CD11c^+^ cells compared to control mice, suggesting that IL‐4Rα‐expressing CD11c^+^ cells play an important role in the development of adaptive immune responses during schistosomiasis. As CD11c can be expressed not only on DCs but also on various other myeloid cell subsets during inflammatory diseases, identifying which CD11c^+^ myeloid cells are involved in driving optimal adaptive immune responses and supporting liver fibrogranulomatous inflammation during schistosomiasis would be useful for developing novel control strategies.

## 4 METHODS

### 4.1 Generation and genotyping of CD11c^cre^IL‐4Rα^−/lox^ BALB/c mice

CD11c^cre^ mice were backcrossed to a BALB/c background for 9 generations, then intercrossed with IL‐4Rα^–/–^ BALB/c mice.^24^ These mice were further mated with homozygous IL‐4Rα^lox/lox^ BALB/c mice to generate hemizygous CD11c^cre^IL‐4Rα^–/lox^ BALB/c mice. Hemizygous (IL‐4Rα^–/lox^) mice were used as wild‐type littermates controls in all experiments. Mice were genotyped as described previously.^24^, ^37^ All mice were housed in specific pathogen‐free barrier conditions in individually ventilated cages at the University of Cape Town biosafety level 2 animal facility. Experimental mice were age and sex matched and used between 8–12 wk of age.

### 4.2 Ethics statement

This study was performed in strict accordance with the recommendations of the South African national guidelines and University of Cape Town practice of laboratory animal procedure. All mouse experiments were performed according to the protocols approved by the Animal Research Ethics Committee of the Faculty of Health Sciences, University of Cape Town (protocol number: 010/041). Efforts were made to minimize and reduce suffering of animals.

### 4.3 Live *S. mansoni* infection of mice

Mice were percutaneously infected with 100 live *S. mansoni* cercariae that were provided by the Schistosome Research Reagent Resource Center for distribution by BEI Resources, (NIAID, NIH, USA: *Schistosoma mansoni*, Strain NMRI Exposed *Biomphalaria glabrata*, Strain NMRI, NR‐21962. Mice were monitored weekly until the endpoint was reached (7 wk postinfection).

### 4.4 Footpad model

Mice were challenged by injection of 2,500 *S. mansoni* eggs into the right hind footpad and killed 7 d postchallenge. *S. mansoni* eggs were purchased from the Theodor Bilharz Research Institute (Schistosome Biological Supply Center, Egypt) and stored at –80°C until use. The integrity and viability of the eggs were evaluated using a light microscope prior to use.

### 4.5 Flow cytometry

The following Abs were used for flow cytometry: CD19‐PerCP Cy5.5, CD4‐PE, CD11c‐APC, CD11b‐PE, MHCII‐FITC CD62L‐APC, and CD44‐FITC (BD Bioscience, Erembodegem, Belgium). Activation profile of CD11c^+^MHCII^+^ and CD11b^+^MHCII^+^ was analyzed by flow cytometry. Intracellular expression of iNOS was detected using rabbit anti‐mouse iNOS Ab (Abcam Cambridge, United Kingdom) with goat anti‐rabbit PE (Abcam Cambridge, United Kingdom). Intracellular Ym‐1 expression was detected using Ym‐1‐Biotin with Strep‐APC. Staining specificity was verified by appropriate isotype matched Ab controls. Cells were acquired on a FACSCalibur machine (BD Immunocytometry system, San Jose, CA) and data were analyzed using Flowjo software (Treestar, Ashland, OR).

### 4.6 Intracellular cytokine secretion staining

For detection of intracellular cytokines, MLN cells from *S. mansoni*‐infected mice or pLN from *S. mansoni* eggs‐injected mice were plated at 2 × 10^6^ cells/well and stimulated at 37°C for 4 h with 50 ng/ml phorbol (myristate acetate), 250 ng/ml Ionomycin, and 200 μM monensin in IMDM/10% FCS (all purchased from Sigma–Aldrich). Cells were stained with extracellular markers (CD4 Biotin‐APC or CD19 PercP), fixed for 30 min on ice in 2% (w/v) paraformaldehyde, and permeabilized with 0.5% saponin buffer and stained with PE‐labelled anti‐mouse IL‐4, IL‐5, IL‐13, and IFN‐γ for 30 min. Acquisition was performed using a FACSCalibur (BD Immunocytometry Systems) and data were analyzed using FlowJo software (Treestar).

### 4.7 Cell preparation and ex vivo restimulation

Single cell suspensions were prepared by pressing the draining lymph nodes through 70 μM cell strainers. Cells were resuspended in complete IMDM (Gibco Walthan, MA, USA) supplemented with 10% FCS (Gibco) and penicillin and streptomycin (100 U/ml and 100 μg/ml, Gibco). The cells were cultured at 2 × 10^6^ cells/ml in 48‐well plates coated with α‐CD3 (20 μg/ml) or soluble egg Ag (SEA, 20 μg/ml) and incubated at 37°C in a humidified atmosphere containing 5% CO_2_. Supernatants were collected after 72 h and cytokines were measured by ELISA. Quantities of IL‐4, IL‐5, IL‐10, IL‐13, and IFN‐γ were measured by sandwich ELISA as previously described.^37^

### 4.8 Enzyme‐linked immunosorbent assays

Cytokines in supernatants were measured by sandwich ELISA as previously described.^37^ For detection of Ab isotypes, blood was collected in serum separator tubes (BD Bioscience, San Diego, CA) and serum was separated by centrifugation at 8,000 × *g* for 10 min at 4°C. Titers of SEA‐specific IgG1, IgG2a, IgG2b, and total IgE were determined as previously described.^37^

### 4.9 Hydroxyproline assay

Hydroxyproline content as a measure of collagen production was determined using a modified protocol.^38^ Briefly, weighed liver samples were hydrolyzed and added to a 40 mg Dowex/Norit mixture. The supernatants were neutralized with 1% phenolphthalein and titrated against 10 M NaOH. An aliquot was mixed with isopropanol and added to chloramine‐T/citrate buffer solution (pH 6.5). Erlich's reagent was added and absorbance was read at 570 nm. Hydroxyproline levels were calculated using 4‐hydroxy‐L‐proline (Calbiochem, San Diego, CA, USA) as a standard, and results were expressed as μmoles hydroxyproline per weight of tissue that contained 10^4^ eggs.

### 4.10 AST measurement

Serum was sent to the Department of Surgery at the University of Cape Town (Histology facility) to measure AST levels.

### 4.11 Histology

Liver and gut samples were fixed in 4% (v/v) formaldehyde in PBS, embedded in wax, and processed. Sections (5–7 μm) were stained with hematoxylin and eosin (H&E) and analine blue solution (CAB) and counterstained with Wegert's hematoxylin for collagen staining. Micrographs of liver granuloma were captured using a Nikon 5.0 mega pixel color digital camera (DCT DS‐SMc). The diameter of each granuloma containing a single egg was measured with the ImageJ 1.34 software. An average of 25 granulomas per mouse were included in the analyses.

### 4.12 Statistics

Statistical analysis was conducted using GraphPad Prism 4 software. Data were calculated as mean ± sd. Statistical significant was determined using two‐tailed Mann–Whitney nonparametric Student's *t*‐test, one‐way or two‐way ANOVA with Bonferroni's posttest, defining differences to IL‐4Rα^−/lox^ mice as significant (*P ≤ 0.05; **P ≤ 0.01; ***P ≤ 0.001). (Prism software; http://www.prism-software.com).

## Supporting information

Supporting Information

Supporting Information

Supporting Information

Supporting Information

Supporting Information

Supporting Information
