# Supplementary figures and images for: IL‐4Rα‐expressing CD11c+ cells contribute to driving optimal cellular responses during Schistosoma mansoni infection in mice
Source: J Leukoc Biol. 2018 Nov 30;105(2):307–16. doi: 10.1002/JLB.MA0318-115R (PMC6391868; doi:10.1002/JLB.MA0318-115R)

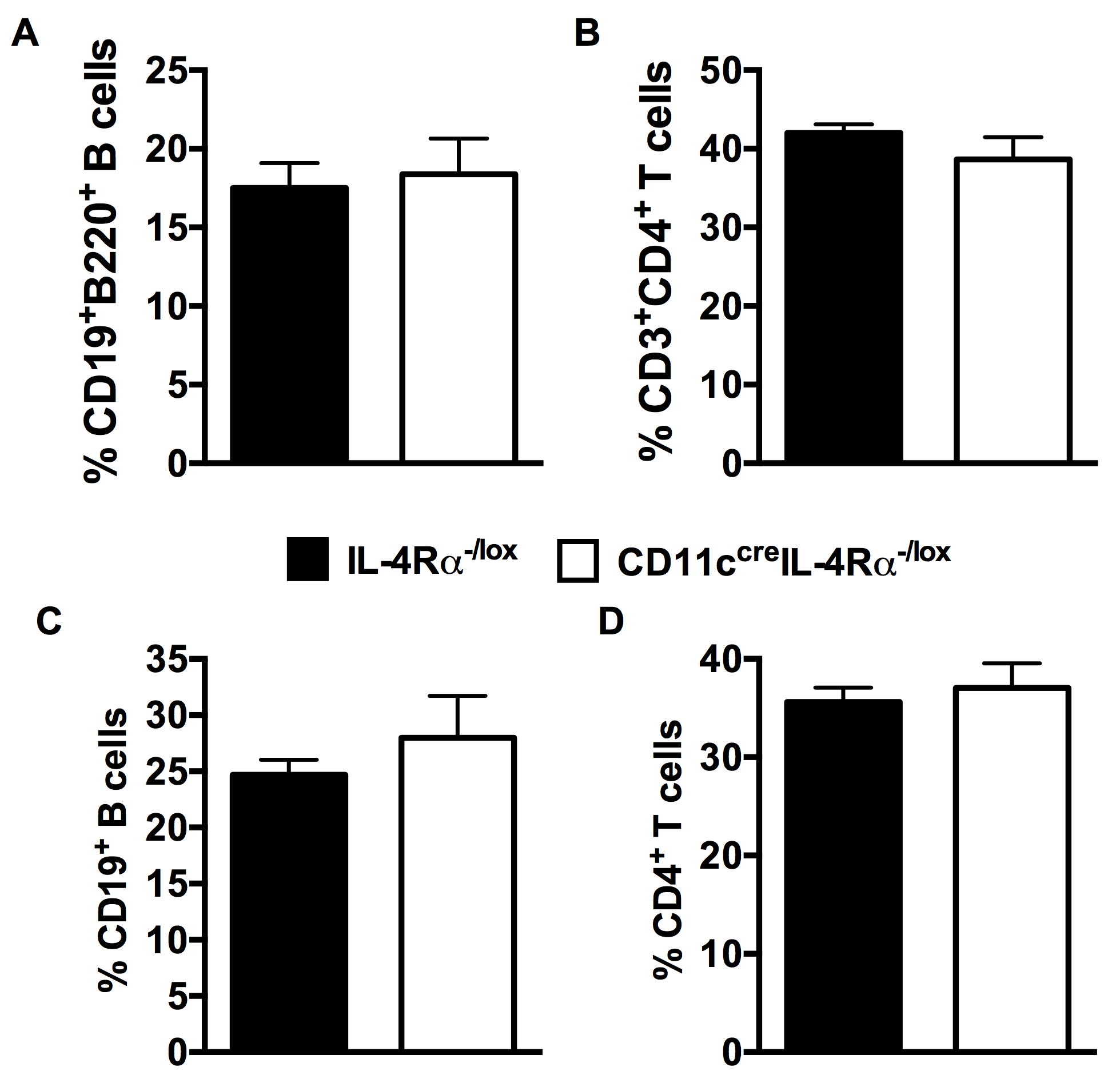

Supplement: Supplementary file 1 — Supporting Information [file JLB-105-307-s001.tiff]

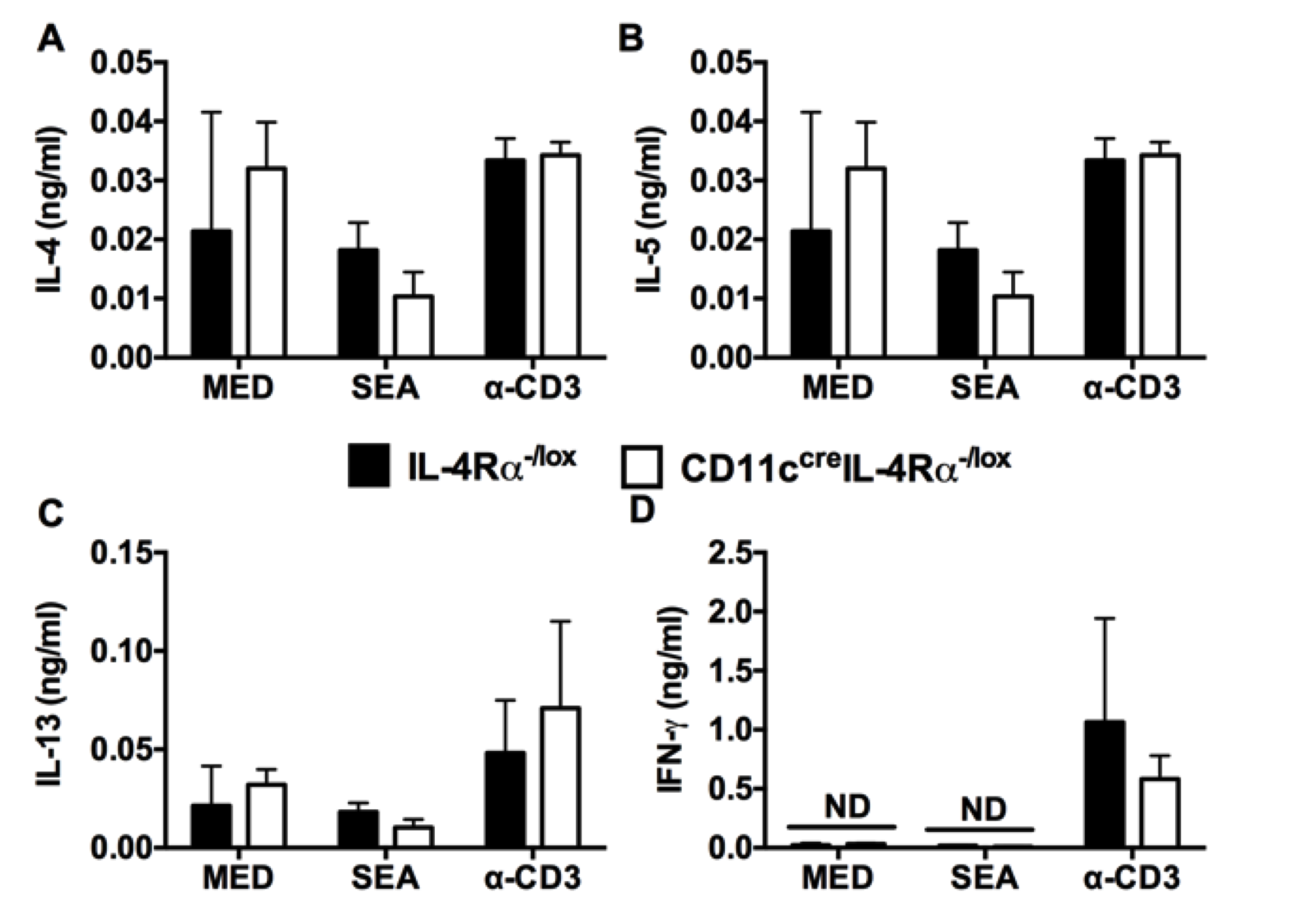

Supplement: Supplementary file 2 — Supporting Information [file JLB-105-307-s002.tiff]

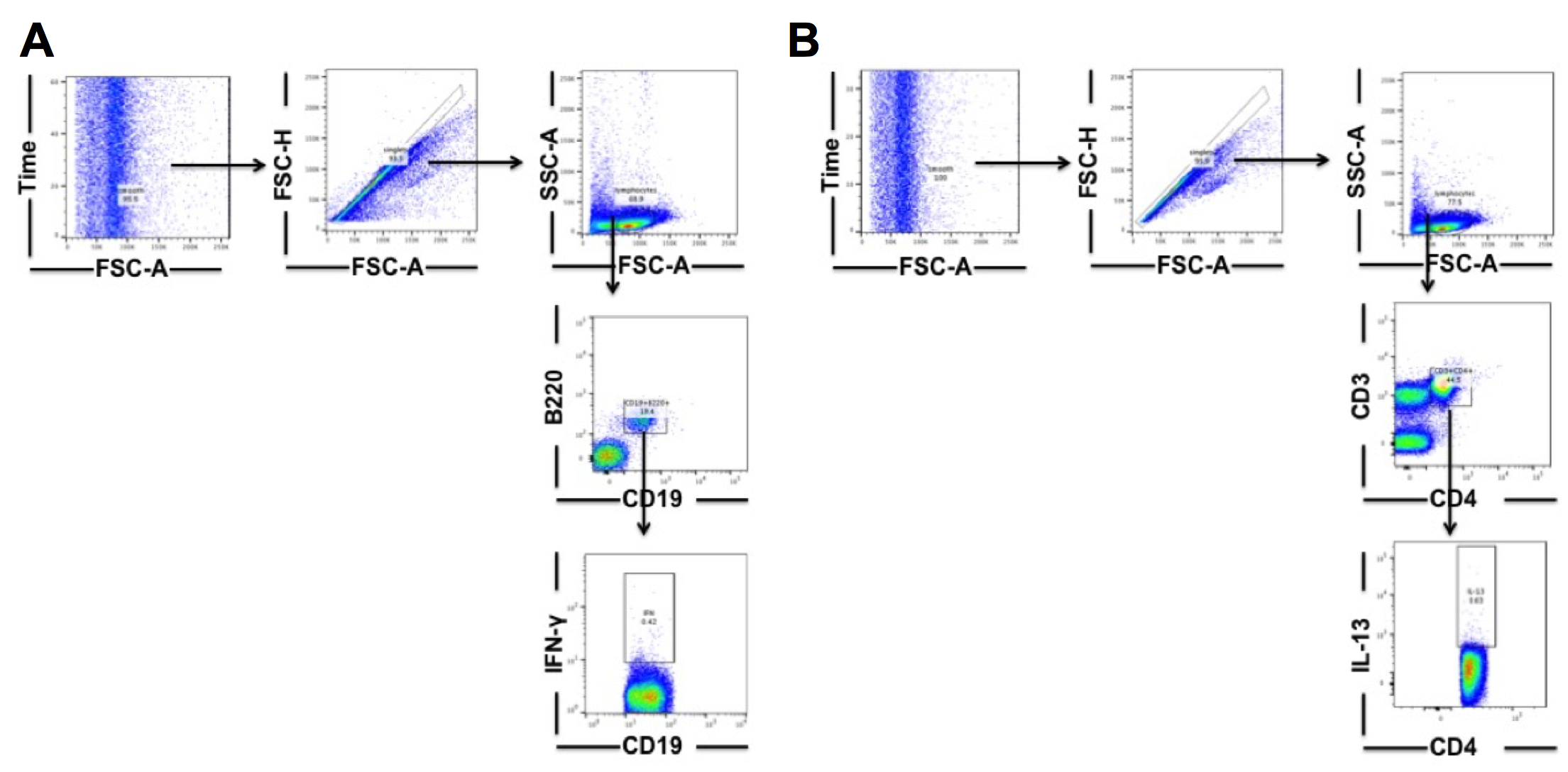

Supplement: Supplementary file 3 — Supporting Information [file JLB-105-307-s003.tiff]

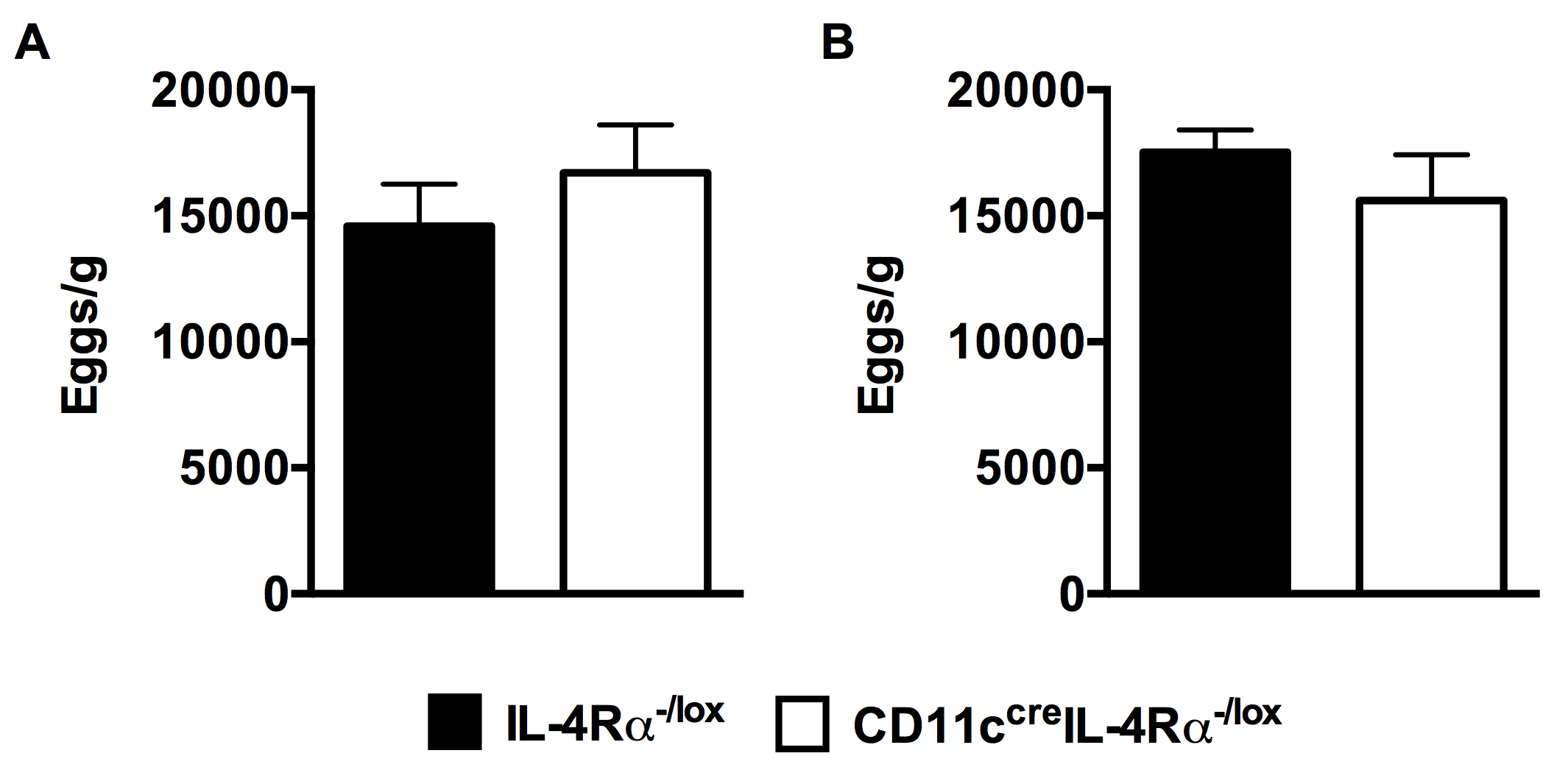

Supplement: Supplementary file 4 — Supporting Information [file JLB-105-307-s004.tiff]

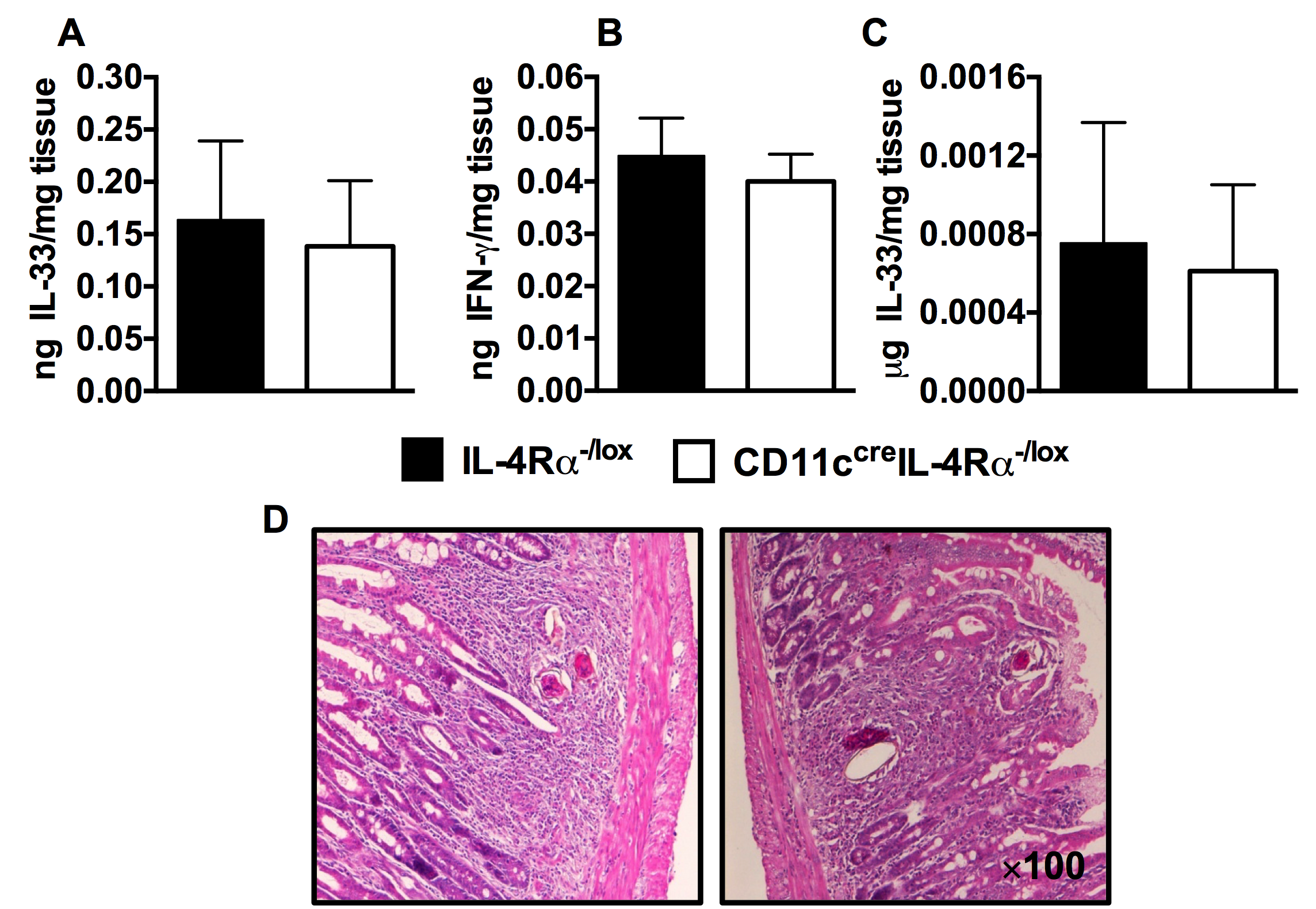

Supplement: Supplementary file 5 — Supporting Information [file JLB-105-307-s005.tiff]

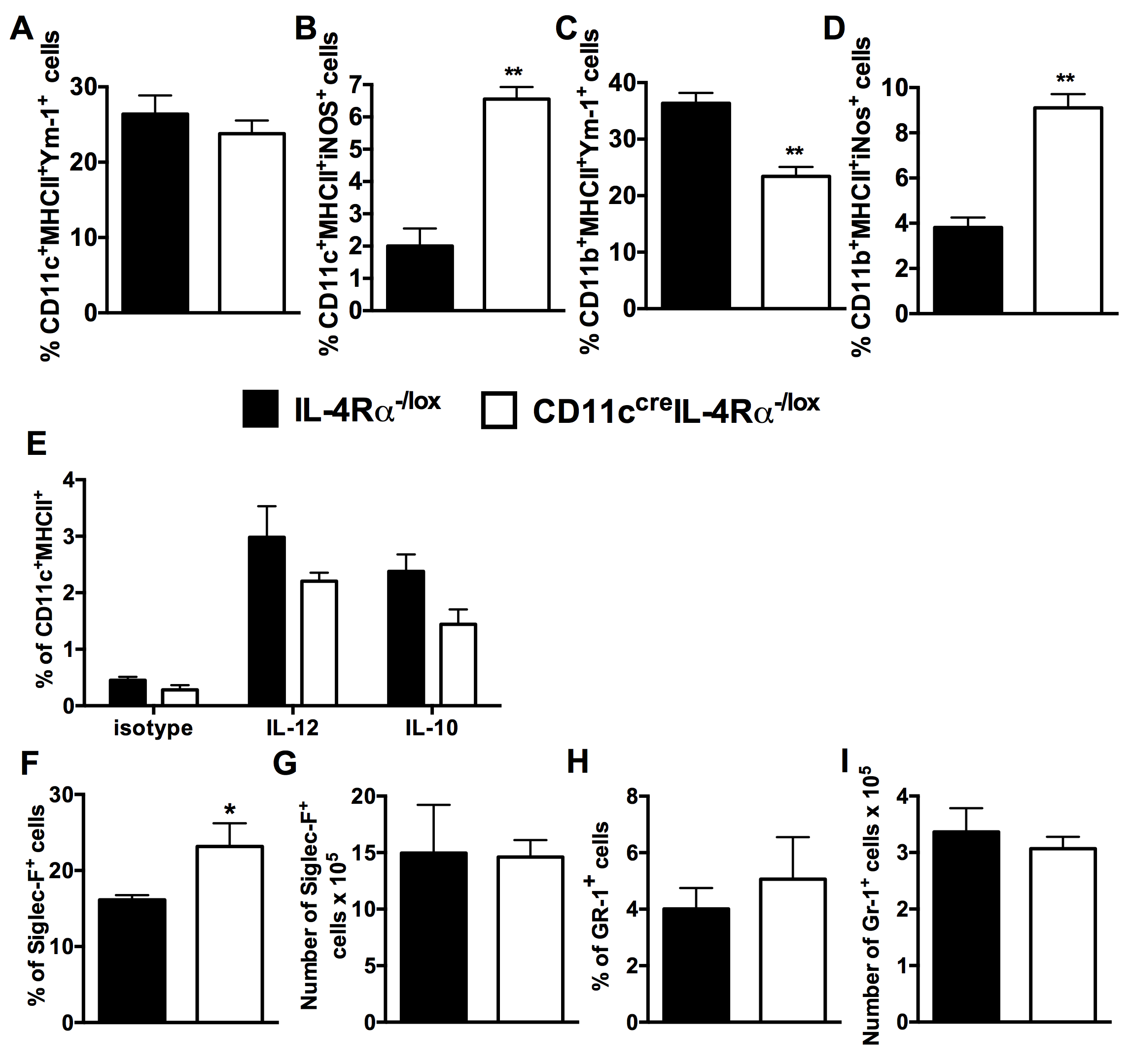

Supplement: Supplementary file 6 — Supporting Information [file JLB-105-307-s006.tiff]
